# Pedestrian attribute recognition using two-branch trainable Gabor wavelets network

**DOI:** 10.1371/journal.pone.0251667

**Published:** 2021-06-01

**Authors:** Imran N. Junejo

**Affiliations:** College of Technological Innovation, Zayed University, Dubai, U.A.E.; Taipei Medical University, TAIWAN

## Abstract

Keeping an eye on pedestrians as they navigate through a scene, surveillance cameras are everywhere. With this context, our paper addresses the problem of pedestrian attribute recognition (PAR). This problem entails recognizing attributes such as age-group, clothing style, accessories, footwear style etc. This multi-label problem is extremely challenging even for human observers and has rightly garnered attention from the computer vision community. Towards a solution to this problem, in this paper, we adopt trainable Gabor wavelets (TGW) layers and cascade them with a convolution neural network (CNN). Whereas other researchers are using fixed Gabor filters with the CNN, the proposed layers are learnable and adapt to the dataset for a better recognition. We propose a two-branch neural network where mixed layers, a combination of the TGW and convolutional layers, make up the building block of our deep neural network. We test our method on twoo challenging publicly available datasets and compare our results with state of the art.

## Introduction

Pedestrian attribute recognition (PAR) is one of the active areas of research in computer vision. PAR problem deals with identifying a number of visual attributes from a pedestrian image data. The identified attributes can belong to different classes, e.g. clothing style, footwear, gender, age group etc. A successful outcome of this research can be applied to various domains. It can be employed for motion analysis [1], where it can be used to identify crowd behavior attributes. Another important area of application is image-based surveillance or visual features extractions for person identification [2, 3]. Other applications include video analytics for business intelligence, or searching a criminal database for suspects using the identified visual attributes. One of the main factors that makes this problem very difficult is the varying lighting conditions. Attributes of the same type of clothing can appear completely different under dynamic lighting conditions. For example, distinguishing black color from a dark blue colors is very difficult in certain weather conditions. These colors will appear very similar to the camera in a darker environment. Occlusion also complicates the visual attribution identification and recognition. These occlusions can be either complete or partial and can result from camera orientation or from object self occlusions. Thus if a person wears a hat, it might appear partially in an image, or its shape might be completely different in another image. Similarly, the orientation of a person or a camera can hide a backpack, for example, partially or completely from a particular view. These examples clearly show that the setup of an acquisition environment for image or video capture results in a high intra-class variations for the same visual attributes.

**Fig 1:**
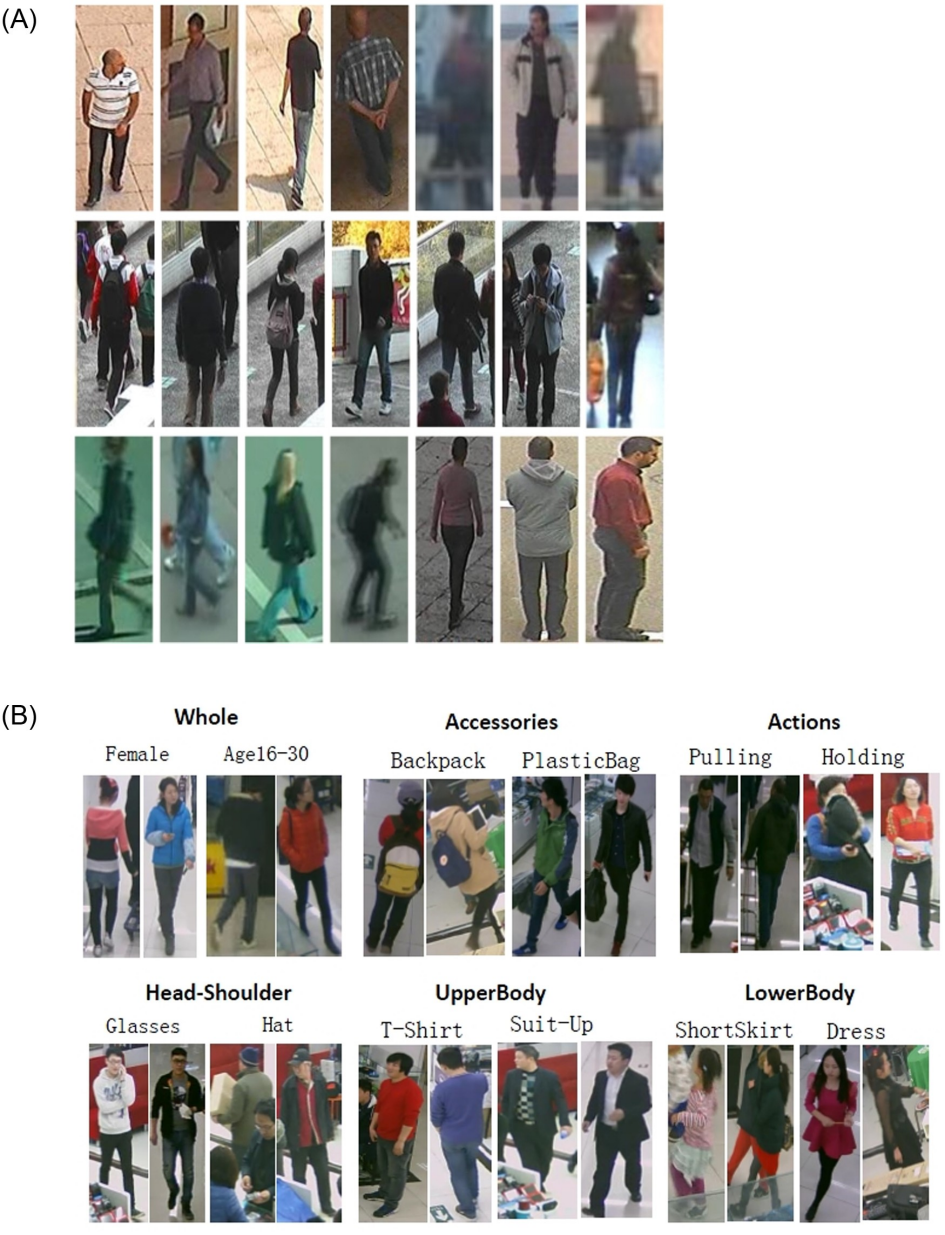
(a) PETA [4] dataset Samples. (b) RAP [5] dataset samples.

The distance of an object from the camera affects how that object appears in an image. If the object is very far from the camera, or if the image resolution is very low, any visual attribute, e.g. dress, hat, backpack, scarf, shoes etc. will only occupy a few pixels in the image. The combination of low image resolution, in addition to the self-occlusions or view-oriented occlusions, makes visual attribute identification a very challenging problem. Many of these issues can be seen in the most widely used pedestrian datasets. Fig 1 shows some of the samples from the PEdesTrian Attribute (PETA) [4] and A Richly Annotated Pedestrian (RAP) [5] datasets. PETA is the largest benchmark dataset. It comprises of 19000 images of different resolution that cover more than 60 attributes. The dataset is acquired from real-world surveillance camera systems and includes images of 8, 705 persons. It is a very challenging dataset because of the acquisition setup and scene settings. As can be seen in Fig 1, the quality of images is very low as well. This is due to a number of factors: images are very low resolution, acquisition problem results in a significant blur, and many of the attributes are hidden due to severe occlusions. RAP dataset comprises of 41 thousand images covering 72 attributes and is acquired from multiple viewpoints. The dataset shows a large variation in the attributes due to pedestrian appearance, viewpoints and severe occlusions. After analyzing these datasets, it is observed that visual attributes identification from these images is a difficult task due to the very low quality of the images. Many of the attributes are not completely visible due to occlusions. Moreover, due to the fast motion or acquisition problems, some of the objects appear quite blurred thus making it a very challenging problem.

Visual attribute recognition problem can be solved in different ways, but the predominant solutions involve a two step process. In the first step, a feature extraction algorithm is employed to find a feature representation of the attributes. A large number of feature extraction solutions are discussed in the computer vision literature. Most of these techniques require a very expert domain knowledge, and also needs a very high level of fine tuning for an accurate representation of visual attributes. For feature representation, methods like SIFT [6], HoG [7] or Haar-like features [8] have been employed in the field rigorously over the past many years. Feature extraction is followed by the attributes classification step. For classification, Support Vector Machines (SVM) [4] has been the most widely used technique in the last decade.

In recent years, the convolutional neural networks (CNNs) have almost completely replaced SVMs for classification tasks. Compared to earlier attribute learning or image classification methods, CNNs are more effective and robust. In this work, for PAR, we introduce the use of the Gabor wavelets, which have been used in the computer vision literature extensively over the last few decades. However, there have been only few works that use the Gabor wavelets in conjunction with the CNNs. For majority of the works that do employ these wavelets, the filters are pre-constructed and then fed to the convolutional network. However, we adopt an approach where the convolutional network is employed to learn the wavelet parameters along with learning the dataset. These Trainable Gabor wavelets (TGW) [9] make up for the backbone of our network. Each TGW accepts a single channel input, with a multi-channel output, and learns the best parameters to generate a set of Gabor filters. TGW layer contains a 1 × 1 convolution layer that uses the steerability of Gabor wavelets to address orientation issues. We also use a regular convolutional layer to extract features from the input as well. These outputs from TGW and convolution layers are stacked together, refer to as mixed-layer, and make up the building blocks of our network. The proposed network, shown in Fig 3, takes a color image as input and passes it through two branches of the network. Both the top and the bottom branch contains equal number of mixed-layers. The set of parameters for each mixed-layer are different. Each branch consists of three mixed-layers. The output of these layers are stacked and passed through a series of convolution layers. A fully connected (fc) layer connects to the final network output layer. The proposed network architecture is simple and is trainable with a standard gradient-decent method.

Our main contributions are:

- We for the first time introduce the use of trainable Gabor wavelets to the problem of pedestrian attribute recognition.
- We propose a novel two-branch network that, while learning the Gabor wavelets parameters, combines the wavelet features with the regular convolution layers.
- The proposed method is demonstrated to have better recognition results than state of the art on two of the most challenging public datasets.

The remainder of the paper is organized as follows: a detailed discussion on the state of the art is presented in the **Related Work** section, and the section **Main Approach** presents the main content of our proposed method. We report on the various datasets and the associated results are mentioned in the section **Evaluation**, before our final remarks in the **Conclusion**.

## Related work

In this section we will discuss the works that are related most closely to our method, a detailed survey can be found here [10]. PETA [4] is one of the most widely used pedestrian datasets. While introducing the dataset, the Deng et al. [4] used the luminance channel and applied Ensemble of Localized Features (ELF) and Gabor and Schmid filter on it. To address the class imbalance problem they also applied ikSVMs [11] on each attribute separately. They also proposed using the Markov Random Field (MRF) to exploit the context from neighboring images. In their representation, each image is a node and the link between two nodes is determined by the similarity between the images. RAP dataset [5] is acquired from multiple viewpoints that introduces significant variations for the same attributes along with severe occlusions. They employed two CNN models based on Caffe framework [12] to analyze the impact of the variations introduced by different viewpoints and occlusions on the overall classification of the attributes. They trained SVMs in addition to the adopting of ELF. Additionally, they divided the image into multiple blocks (three in their case) to employ a part-based classification scheme. For their work, the parts were comprised of: upper body (torso), lower body, and head and shoulders. Joo et al. [13] proposed another approach that also employed part-based recognition. In their work, they first crated Histogram of Oriented Gradient (HoG) features from an image subdivided into multiple overlapping regions. For the attributes classification, they employed a Poselet-based approach [14]. Zhao et al. [15] proposed a solution that employed a Recurrent Neural Network (RNN). The authors proposed an end-to-end Recurrent Convolutional (RC) and Recurrent Attention (RA) models. RC model mines the correlations among different attribute groups, while the intra-group attention correlation and intra group spatial locality is used by the RA model to improve the performance and robustness of pedestrian attribute recognition. However, their network has a deep architecture, hence the number of parameters is quite large. In another part-based approach, Zhu et al. [16] proposed a CNN-based solution where the human body is divided into 15 parts, and a CNN is trained separately for each part. The contribution of each attribute determines the weight of the corresponding CNN. Zhou et al. [17] first extracted mid-level features from detection layers using GoogLeNet. They localized the pedestrian attributes by fusing and clustering the activation maps of the detection layers. Only the image labels are used to train the detected layers in order to learn the relationship between the mid-level features and the pedestrian attributes. For training a max-pooling based weakly-supervised object detection technique is employed. Chen et al. [18] proposed a part-based network that combined LOMO features [19] with CNN extracted features. They showed that the Scale-Invariant Local Ternary Patterns and HSV histograms based LOMO features are illumination-invariant texture and color descriptors. Li et al. [20] used pedestrian body structure knowledge and proposed a pose-guided model. In the first step, the model computes the transformation parameters to estimate the pose from the image. Based on the pose information it then localizes the body parts. Final attribute recognition is estimated by fusing multiple features. Another parts localization method is proposed by Liu et al. [21]. They proposed a Localization Guide Network (LGNet) that uses a CNN model based on Inception-v2 [22] for feature extraction. Afterward, a global average pooling layer (GAP) is adopted to extract global features. The fusion of global and local features is used to obtain the pedestrian attributes classification. Li et al. [23] presented a visual semantic graph based approach that used ResNet-50 to for the pedestrian images feature extraction. Junejo et. al. [24] also presented a multi-branch approach using different color space input. The proposed network contains a large number of parameters because it had more than fifty layers.

Sarfraz et al. [25] proposed an end-to-end CNN-based network (VeSPA). This network had four parts, where each part corresponds to a specific pose category. Pose-specific attributes of each category are learned by each of these network parts. Their work demonstrated that coarse body pose information greatly influences the pedestrian attribute recognition. They extended their work in [26] and added a ternary view classifier in a modified approach that employed a global weighting solution. In this work, the global weighting solution for feature maps was employed before the final embedding. P-Net [27] employs a part-based approach. Based on GoogLeNet, the method guides the refined convolutional feature maps to capture different location information for the attributes related to different body parts. A joint person re-identification and attribute recognition approach (HydraPlus-Net) is presented by Liu et al. [28]. HydraPlus-Net is an Inception-based network and aggregates feature layers from multi-directional attention modules for the final feature representation. Sarafianos et al. [29] presented a multi-branch network that employed a simple weight scheme to address the class imbalance problem. They extracted visual attention masks to guide the network to crucial body parts. The masks are then fused at different scales to obtain a better feature representation. Another end-to-end method for person attribute recognition that uses Class Activation Map (CAM) network [30] to refine attention heat map is proposed by Guo et al. [31]. The heat map identifies the areas of different image attributes. They use CAM network to refine the attention heat map for an improved recognition. A Harmonious Attention CNN (HA-CNN) based joint learning approach for person re-identification is presented in [32]. They used HA-CNN for the joint learning of hard regional attention and soft pixel attention. Feature representation is obtained by this simultaneous optimization. A Multi-Level Factorization Net (MLFN) that factors the visual appearance of a person into latent discriminative factors is proposed by [33]. The factorization is done without manual annotation at multiple semantic levels. A Transferable Joint Attribute-Identity Deep Learning (TJ-AIDL) model that allows for a simultaneous learning of an identity discriminative and attribute-semantic feature representation is proposed by [34]. Si et al. [35] proposed a Dual ATtention Matching network (DuATM), which is a joint learning end-to-end person re-identification framework. Their method simultaneously performs context-aware feature sequences learning and attentive sequence comparison in a joint learning mechanism for person re-identification.

A Generative Adversarial Network based pose-normalized person re-identification framework is presented in [36]. They learn pose invariant deep person re-identification features using synthesized images. A deep CNN based method to learn partial descriptive features for efficient person feature representation is presented in [37]. They employed a pyramid spatial pooling module and reported an improvement of 2.71% on the PETA dataset over [25]. [38] improved over [25] by employing a deeper network based on a context sensitive framework. The proposed network improved generalization and classification accuracy by creating a richer feature sets using deeper residual networks (ResNet) and achieved the best in class results on attribute recognition datasets. [39] presented a visual semantic graph reasoning framework that modeled spatial and attribute relationships using two types of graphs. For reasoning, they employed Graph Convolutional Network that encapsulates the spatial relationship between local regions of the image and the potential semantic relationship of the attributes. [40] used Recurrent Attention (RA) and Recurrent Convolutional (RC) to present a dual model approach for pedestrian recognition. The RC model employed a Convolutional-LSTM model to establish the correlations between the different groups of attributes. To improve the overall robustness, the RA model used both local attention correlation and global spatial locality.

Using Gabor wavelets with CNNs have received a tremendous attention as well [9, 41–43]. [41] use a Gabor filter bank as the first layer of a CNN and the bank gets updated using the standard back-propagation network leaning phase. [42] also use Gabor filters in the first layer of the network. While introducing lateral inhibition to enhance network performance, they use a n-fold cross validation to search for the best parameters. Authors in [43] introduce a Gabor Neural Network (GNN) where Gabor filters are incorporated into the convolution filter as a modulation process, in a spirit similar to the above mentioned works. In contrast to the above works where fixed Gabor filters are used [9], introduce a trainable Gabor wavelets (TGW) layer. The authors present a method where the hyperparameters of the wavelets are learned from the input and a novel 1 × 1 convolution layers are employed to create steerable filters. In this paper, we propose using this TGW layer with our proposed CNN for a novel solution to the problem of PAR. We test on two challenging datasets and show a considerable improvement over state of the art.

## Main approach

In this section, we start with the description of Gabor wavelets layer before describing the architecture of our network in general.

### Gabor wavelets layer

**Fig 2:**
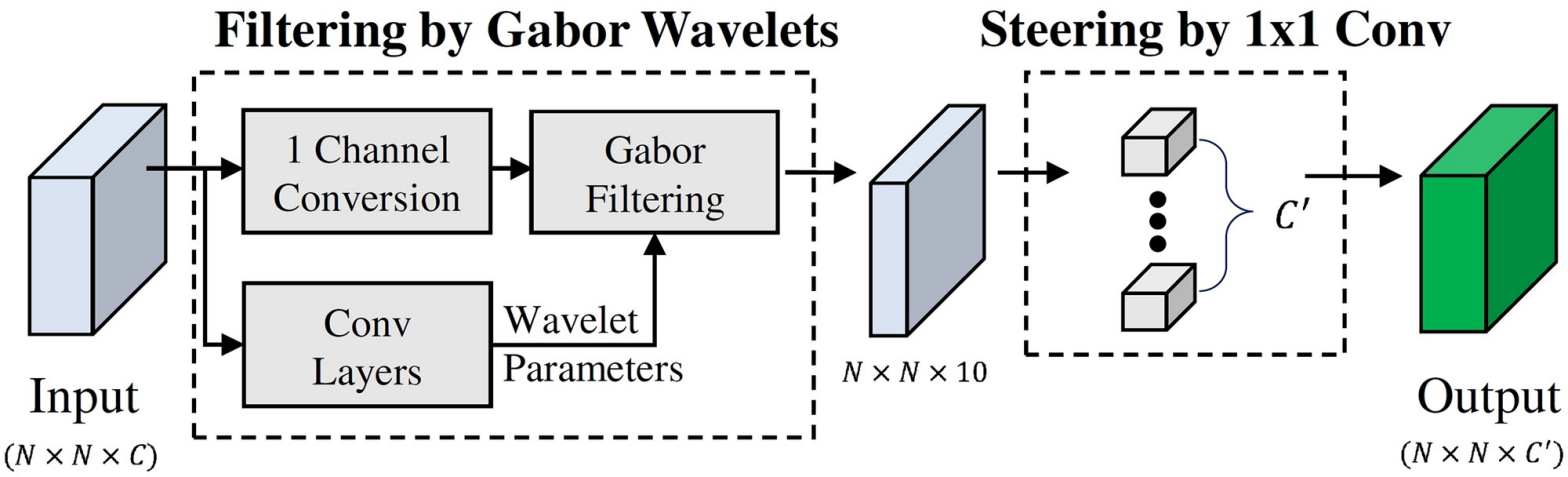
Trainable Gabor Wavelets (TGW) layer [9]: Inputs and outputs are multichannel. A neural network is used to generate Gabor wavelets hyperparameters. These generated Gabor filters are then applied to the input. 1 × 1 convolution layer is added to enable the steerability of the Gabor wavelets.

We make use of the Trainable Gabor wavelets (TGW) layer as proposed by Kwon et. al. [9] (see. Fig 2). A neural network is used to generate the hyperparameters for the Gabor wavelets and the generated Gabor filters are applied to filter the inputs. In order to capture the essential input features, a 1 × 1 convolution layer is added to the TGW layer to capture features at different orientations.

#### Hyperparameter estimation

The 2D Gabor wavelets can be described as:

$$\begin{array}{c} G\left(x,y\right)=\exp(-\frac{X^{2}+\gamma Y^{2}}{2\sigma^{2}})\times \cos(\frac{2\pi }{\lambda }X) \end{array}$$ (1)

where *γ* represents aspect ratio, λ represents wavelength of the sinusoidal, *σ* represents width or the standard deviation, *X* = *x*cos(*θ*) + *y*sin(*θ*), *Y* = −*x*sin(*θ*) + *y*cos(*θ*), and *θ* is an angle in the range [0, *π*]. Thus in order to specify a continuous Gabor wavelets, we need to determine the set of hyperparameters {*γ*, *θ*, λ, *σ*}. In order to convert the continuous filter to a discrete one, a sampling grids need to be defined, which is largely linked to *σ*. A new parameter is thus introduced to compute the discrete filter:

$$\begin{array}{c} G\left[m,n\right]=g\left(u,v\right)=(\frac{m}{\left\lfloor \zeta \right\rfloor }\times \zeta ,\frac{n}{\left\lfloor \zeta \right\rfloor }\times \zeta ) \end{array}$$ (2)

where *m* and *n* are in the interval −⌊*ζ*⌋, ⌊*ζ*⌋ + 1, …, ⌊*ζ*⌋, and by just varying ⌊*ζ*⌋, variety of sampling grids can be achieved [9]. For a loss function *L*, we need to compute $\frac{\partial L}{\partial \zeta }$ in order to train for the wavelets layer that is cascaded with our CNN. In order to train for the *ζ*, what remains is to compute $\frac{\partial G[m,n]}{\partial \zeta }$, as $\frac{\partial L}{\partial G[m,n]}$ is handled automatically by the deep learning libraries:

$$\begin{array}{c} \frac{\partial G[m,n]}{\partial \zeta }=\frac{\delta g(u,v)}{\partial u}\frac{\partial u}{\partial \zeta }+\frac{\partial g(u,v)}{\partial v}\frac{\partial v}{\partial \zeta } \end{array}$$ (3)

$$\begin{array}{c} =\frac{\delta g(u,v)}{\partial u}\frac{u}{\zeta }+\frac{\partial g(u,v)}{\partial v}\frac{v}{\zeta } \end{array}$$ (4)

as $\frac{d}{d\zeta }\left\lfloor \zeta \right\rfloor =0$. The remaining parameters $\frac{\partial G[m,n]}{\partial \sigma }$, $\frac{\partial G[m,n]}{\partial \gamma }$, $\frac{\partial G[m,n]}{\partial \lambda }$ can be computed in a similar way and a similar parameterization can be adopted for the parameters *σ*, *γ* and λ.

A very significant parameter for the Gabor wavelets is the orientation (*θ*) parameter. These values are mostly chosen empirically. This parameter is also made trainable to better design orientations for the task at hand. To use the steering property, where a linear combination of finite set of responses can be used to represent convolution at any orientation, a 1 × 1 convolution layer, working as a linear combination layer, is added to the output of the generated filters. For this layer, ten equally spaced fixed orientations are selected, working as basis filters: 9°, 27°, 45°, 63°, 81°, 99°, 117°, 135°, 153°, and 171° [9].

### Attribute recognition network

The above mentioned TGW layer can be thought of as a feature extracting layer. In addition to this, we also employ it as the key building block of our network. Thus, in addition to functioning as the *lowest layer* in the network, it also aids the network in learning high level features.

**Fig 3:**
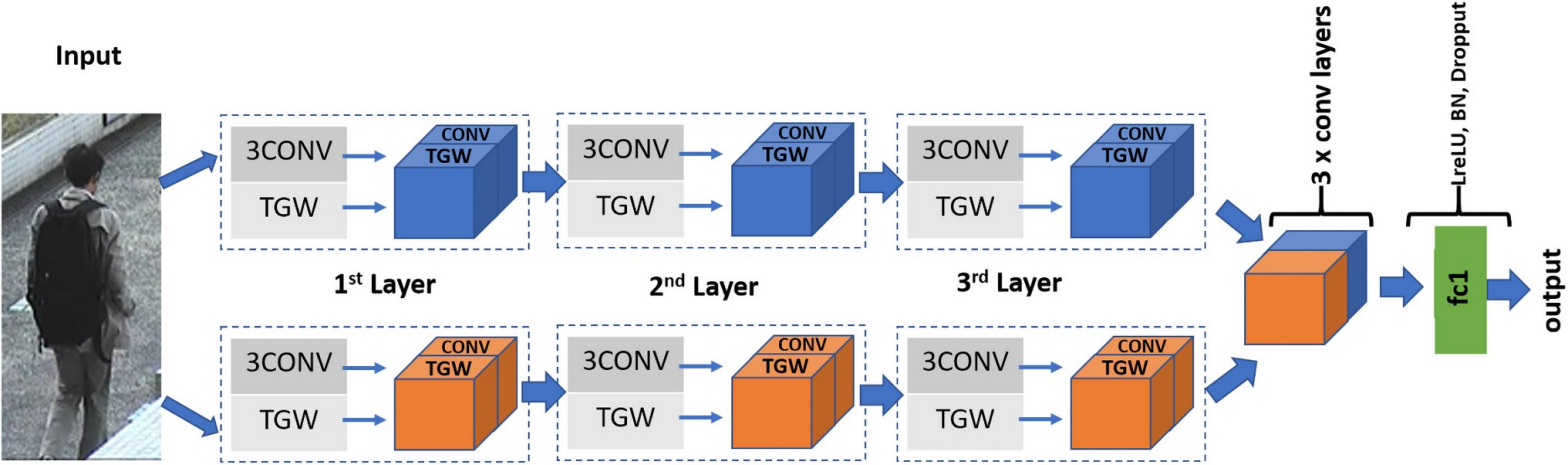
Our approach: The input is passes through two branches in the network. For each branch, the network contains 3 mixed-layers (combination of TGW and 3Conv layer). The output of each branch is followed by three convolutional layers and a fc layer. Size of the last layer of the network matches the number of attributes of the dataset. Parameters of the network are mentioned in Table 1.

The proposed network is shown in Fig 3. A color input image is first converted to a grayscale image. This image is then passed on to the two branches of the network. As can be seen in the figure, each branch consists of 3 mixed-layers. As mentioned above, the mixed-layer is defined as a combination of TGW layer and a 3 × 3 convolution layer. The input to this layer starts with a 1-channel conversion, i.e. a multi-channel input is converted to a 1-channel, which is a summation over the channels operation for all layers except the first layer where we perform a simple color-to-gray image conversion. The top branch (depicted with blue in the figure) and the bottom branch (depicted with orange in the figure) differ only in their parameters, as given in Table 1. The difference in these parameters is clearly visible. The value for (λ_*o*_, *σ*_*o*_, *ζ*_*o*_) in the top branch are larger than the corresponding values in the bottom branch. This intuitively translates into the bottom branch having a smaller receptive field. Thus, smaller or finer features are extracted from the bottom branch whereas the top branch focuses on larger image features.

**Table 1:** Parameters used for the TGW layers.

Layer	*γ*_*o*_	λ_*o*_	*σ*_*o*_	*ζ*_*o*_	TGW Channels	Conv Channels
1 Top	0.3	6.8	5.4	6	64	64
2 Top	0.3	5.6	4.5	5	64	64
3 Top	0.3	4.6	3.6	4	64	64
4 Bottom	0.3	3.5	2.8	3	64	64
5 Bottom	0.3	2.5	2.0	2	64	64
6 Bottom	0.3	2.5	2.0	2	64	64

Each mixed-layer (1 to 3) contains 64 channels from the TGW layer and 64 channels from a 3 × 3 convolution layer (denoted as 3Conv). Thus depth of each mixed-layer output is 128 (concatenation of TGW and 3Conv layer). The network thus contains blocks of layers stacked together. For each 3Conv layer, as the name suggest, the kernel size is of size 3 × 3. Convolution is followed by LeakyReLU activation function, max-pool layer (size 2 × 2), and Batch Normalization (BN) layer. Size of input to each of these stacked layers is, respectively: 227 × 227, 113 × 113, and 56 × 56. As shown in Fig 3, output from the two branches is stacked and follows through three convolutional layers. For each of these layers: kernel size 3 × 3, LeakyReLU activation function, BN layer, a max-pool layer of stride (size 2 × 2).

In order to reduce the number of parameters, the output from the convolution layers are fed into a single fully connected layer (fc1) of size 512. The fc1 layer uses LeakyReLU as the activation function, followed by a dropout layer (*p* = 0.5), to minimize the number of network parameters. The final output layer size matches the number of dataset attributes. Table 2 described these convolutional layers.

**Table 2:** Parameters used for three convolution layers.

Layer	input channels	output channels	kernel size	padding
layer 1	256	256	3 × 3	1
layer 2	256	256	3 × 3	1
layer 3	256	256	3 × 3	1

We have presented a method proposes using Gabor wavelets merged with a deep neural network. Whereas other methods construct Gabor filters manually, our network learns the wavelets parameters, suitable to the dataset. Generated Gabor filters are stacked with convolution layers to build the overall network. As we shall show next, the proposed network is efficient and learns the dataset structure well to perform at par with state of the art.

## Evaluation

Following the channel conversion, the grayscale image is passed through the two branches of the network. Each branch has three mixed-layers in a sequence. Each of the mixed-layer contains an equal number of channels from TGW and 3Conv layer, i.e. 64 and 64. The two branches are stacked together and then three convolution layers are used before a single fully connected layer. LeakyReLU is used as the activation function for all our layers. The output layer uses sigmoid as the activation function.

In order to evaluate our method quantitatively, we compute various measures and report the results below. Although mean accuracy has been widely used in the attribute recognition literature, it however treats each attribute independent of the other attributes. This might not necessarily be the case and an inter-attribute correlation might exist. Therefore, researchers also report *example-based* evaluations, namely accuracy (*Acc*), precision (*Prec*), recall (*Rec*), and F1 score (*F*1) [5].

### Dataset

RAP and PETA are the most widely used datasets for the problem of pattern attribute recognition. Collected from real-time surveillance cameras, the PETA dataset contains 19, 000 images collected from 10 publicly available datasets. The resolution of the images ranges from 17 × 39 to 169 × 365. Collected from a multi-camera setup of around 26 cameras, the RAP dataset contains 41, 585 pedestrian samples. Each attribute is annotated independently and the size of the images range from 36 × 92 to 344 × 554.

Most of the previous works [20, 25] report results on the PETA dataset using only 35 attributes. Similarly, for the RAP dataset, results are reported on 51 datasets. In order to make a fair comparison, we adopt the same scheme and test/train on the same attributes. Similarly, for a fair comparison, experiments are conducted on 5 random splits: we allocate 9, 500 samples for training, 1, 900 samples for validation, 7, 600 samples for testing on the PETA dataset. For the RAP dataset, we split it randomly into 33, 268 training images and 8, 317 test images [25]. We adopted the weighted-cross entropy loss function [20] in order to mitigate the class imbalance problem. Similarly, following other researchers, images are resized to an image resolution of 144 × 48.

#### Pre-processing

Before continuing to the next step, we perform **mean subtraction**: That is, we compute the mean for all images (for each color spaces) and this value is then subtracted from image data. Intuitively for each dimension, this step is equal to centering the data around its origin. Next step involves **normalization**: we compute the standard deviation separately for each color channel and image data is divided by this value.

### Setup

For deep learning, we adopted the KERAS [44] library, which is based on the TensorFlow backend. All experiments were performed on a cluster node with 2 x Intel Xeon E5 CPU, 128GB Registered ECC DDR4 RAM, 32TB SAS Hard drive storage, and 8 x NVIDIA Tesla K80 GPUs.

### Implementation details

We train the network for 50 epochs. LeakyReLU was used as the activation function for all layers of the network. We used Adam as the update optimizer with parameters: learning rate = 1*e*^−4^, *β*_1_ = 0.9 and *β*_2_ = 0.999.

To prevent model over-fitting, we added the dropout layer to the fc layer. We adopt weight decay by a factor of 0.1 after 15 epochs. The batch size was set to be 8. All weights in the network are initialized using He Normal initialization.

For the TGW layers with a steering block, we use the scheme suggested by [45]: we fix the parameters {*γ*, *σ*, λ} as shown in Table 1 while training for *ζ*. This setup yields the best results in our experiments.

### Results

**Fig 4:**
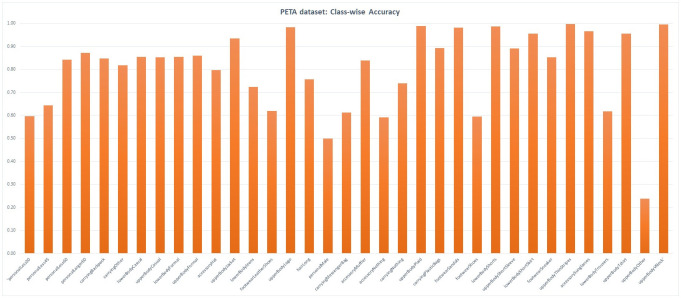
Class-wise accuracy—PETA dataset: The figure shows the obtained class-wise accuracy. The highest accuracy is for the class upperBodyThinStripes. The lowest accuracy is for the class upperBodyOther.

We evaluate the effectiveness of the proposed method on both PETA and RAP datasets. Table 3 shows a comparison of the proposed method with six current state of the art methods. For the PETA dataset, *Acc* obtained from our method is 80.1%. This is higher than all the other methods that we compare with. The obtained results for the other measures (*Pre*, *Rec* and *F*1) is 84.77%, 80.01%, and 81.79% respectively. Class-wise accuracy chart for the PETA dataset is shown in Fig 4. Interestingly, the lowest accuracy is that for the class upperBodyOther. Considering the image resolutions in the dataset, this is indeed a very difficult class to accurately measure. On the other hand, the highest accuracy is that of the classes upperBodyThinStripes and upperBodyVNeck.

**Table 3:** Quantitative results (%) on PETA and RAP datasets. Results are compared with the other benchmark methods. As can be seen, we have comparable results, with considerable improved accuracy for both the datasets.

	PETA [4]				RAP [5]			
	*Acc*	*Prec*	*Rec*	*F*1	*Acc*	*Prec*	*Rec*	*F*1
Chen et. al. [20]	75.07	83.68	83.14	83.41	62.02	74.92	76.21	75.56
Li et. al. [5]	−	−	−	−	63.67	76.53	77.47	77.00
Sudowe et. al. [46]	73.66	84.06	81.26	82.64	62.61	80.12	72.26	75.98
Liu et. al. [17]	74.62	82.66	85.16	83.40	53.30	60.82	78.80	68.65
Sarfaraz et. al. [25]	77.73	86.18	84.81	85.49	67.35	79.51	79.67	79.59
Li et. al. [28]	76.13	84.92	83.24	84.07	65.39	77.33	78.79	78.05
**ours**	**80.1**	84.77	80.1	81.79	**91.5**	**92.59**	**91.6**	**91.9**

**Fig 5:**
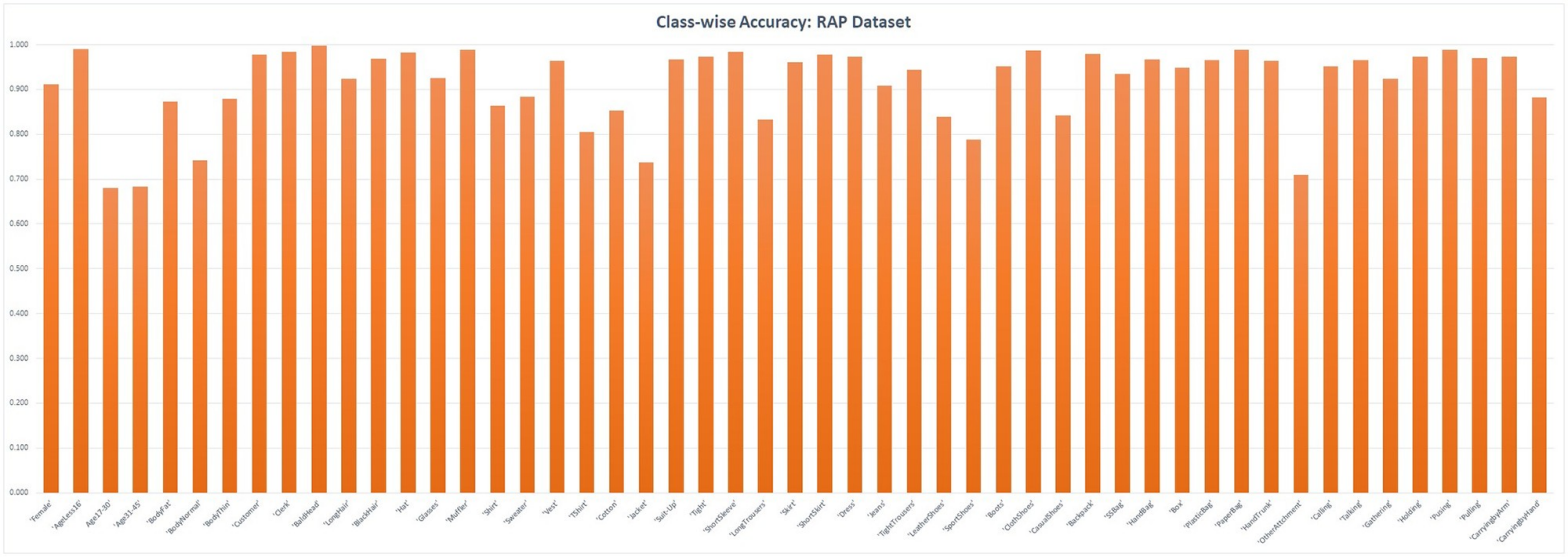
Class-wise accuracy—RAP dataset: The lowest accuracy is that of the classes: Age17–30, Age31–45. The highest accuracy is for the class BaldHead.

For the RAP dataset, similar to the PETA dataset, the obtained results are exceedingly encouraging. The obtained accuracy is 91.5%, while we obtained 92.59% 91.6%, and 91.9% for the remaining measure precision, recall, and F1-score. The obtained results are a considerable improvement over state of the art. One significant reason for this difference is primarily the large size of the RAP dataset. For the RAP dataset, class-wise accuracy is shown in the Fig 5. The class BaldHead is recognized with a highest accuracy score while the two class that had a low score were that of Age17–30, Age31–45. These two classes, naturally, are very difficult to judge, even for experience human observers. Other low performing classes are: Jacket, OtherAttachments.

The proposed method makes a novel use of Gabor wavelets layers. Instead of manually constructing Gabor filters, TGW layers are trainable and are able to correctly estimate model parameters. The paper proposes a two branch network. For each branch, we train three mixed-layers: combination of TGW and 3Conv layers. The output of these branches are stacked and then followed by a fc layer before the final output layer. We have obtained very encouraging results for the key measures. The method is novel and unique in the sense that it does not resort to data augmentation or part-based computations, as employed by [5]. We also do not have to compute pose estimation [20], or construct any hand-crafted features [18]. Our results are an improvement over state of the art and clearly justifies the use of Gabor wavelets layers.

## Conclusion

The works focuses on the problem of Pedestrian Attribute Recognition (PAR). As a novelty, we are introducing trainable Gabor wavelets (TGW) layer to the problem of pedestrian attribute recognition that are cascaded with convolutional layers for an effective learning. The proposed network is two branched where each branch contains three mixed-layers. These mixed-layers are the building blocks of our network; they contain stacked layers from TGW and convolution layers. We have extensively tested the proposed method on the most challenging public datasets and have presented encouraging results. Specifically, we have tested on the PETA and the RAP datasets and demonstrate results (F1-score of 81.79% on PETA and 91.9% on RAP dataset) that are a magnitude improvement over the state of the art. For future work, we intend to further investigate Gabor wavelets for the PAR problem with different network architectures in addition to incorporating 3DCNNs in the proposed architecture.

## Supporting information

S1 Fig (JPG)
