# Supplementary figures and images for: Pedestrian attribute recognition using two-branch trainable Gabor wavelets network
Source: PLoS One. 2021 Jun 1;16(6):e0251667. doi: 10.1371/journal.pone.0251667 (PMC8168894; doi:10.1371/journal.pone.0251667)

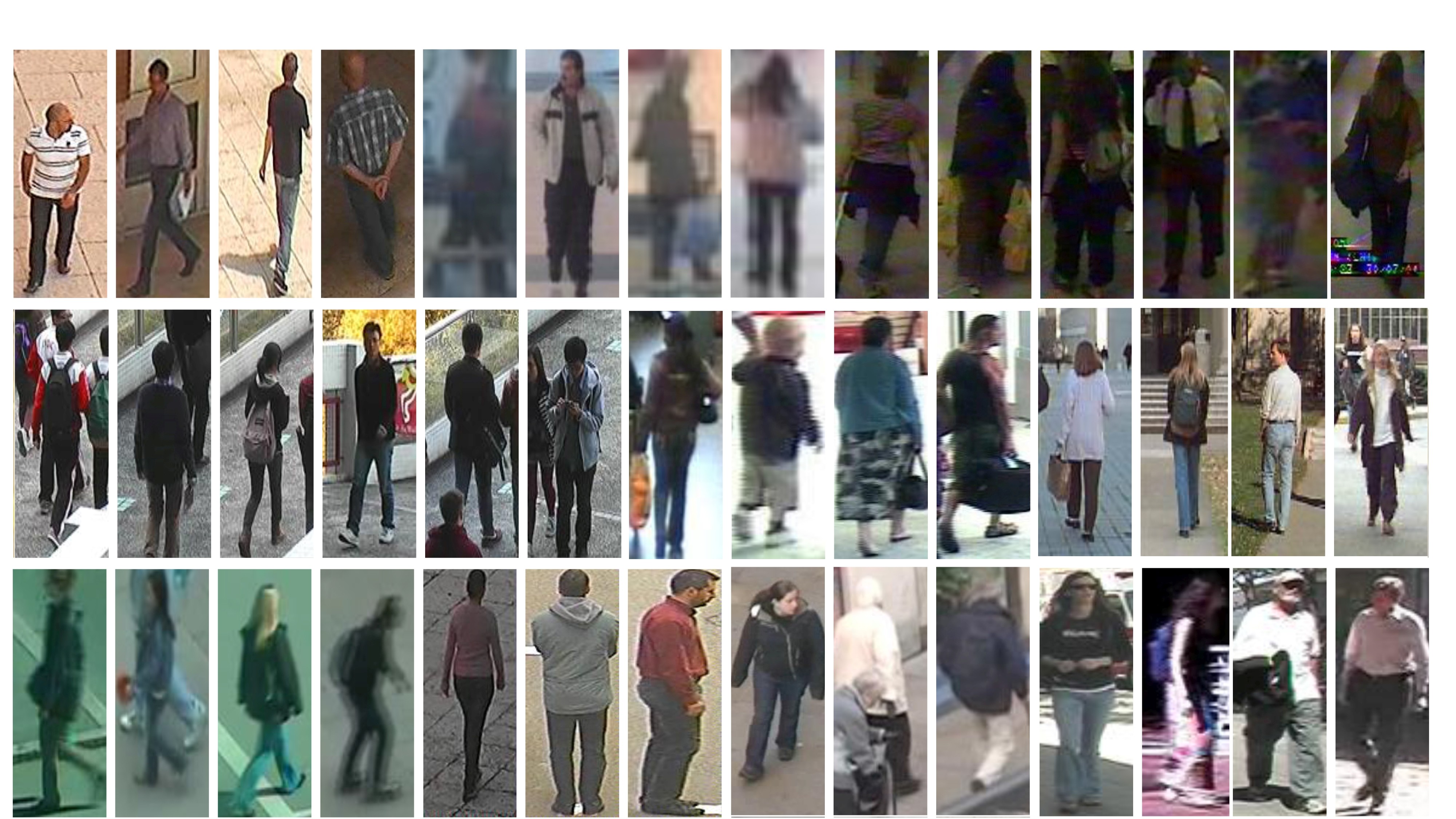

Supplement: S1 Fig — (JPG) [file pone.0251667.s001.jpg]
